# High Prevalence of Neutrophil Cytoplasmic Autoantibodies in Infants with Food Protein-Induced Proctitis/Proctocolitis: Autoimmunity Involvement?

**DOI:** 10.1155/2015/902863

**Published:** 2015-09-21

**Authors:** Alena Sekerkova, Martin Fuchs, Eva Cecrdlova, Veronika Svachova, Ivana Kralova Lesna, Ilja Striz, Helena Tlaskalova-Hogenova

**Affiliations:** ^1^Department of Clinical and Transplant Immunology, Institute for Clinical and Experimental Medicine, 140 21 Prague, Czech Republic; ^2^Immunoflow, 199 00 Prague, Czech Republic; ^3^Laboratory for Artherosclerosis Research, Institute for Clinical and Experimental Medicine, 140 21 Prague, Czech Republic; ^4^Laboratory of Cellular and Molecular Immunology, Institute of Microbiology, ASCR, 142 00 Prague, Czech Republic

## Abstract

*Background.* Food protein-induced proctitis/proctocolitis (FPIP) is the most common noninfectious colitis in children in the first year of life. Along with the overall clinical symptoms, diarrhoea and rectal bleeding are the main manifestations of the disease. There is no routine noninvasive test that would be specific for this type of colitis. The aim of our study was to find a noninvasive laboratory test or tests that may be helpful in differential diagnosis of food protein-induced proctitis/proctocolitis. *Methods.* ANA, ANCA, ASCA, a-EMA, a-tTg, specific IgE, total IgE, IgG, IgA, IgM, and concentration of serum calprotectin were measured in a group of 25 patients with colitis and 18 children with other diagnoses. *Results.* Atypical-pANCA antibodies of IgG isotype were detected in the sera of 24 patients by the method of indirect immunofluorescence, and 5 patients showed also the positivity of IgA isotype. In control samples these autoantibodies were not detected. Other autoantibodies were not demonstrated in either patient or control group. *Conclusions.* Of the parameters tested in noninfectious colitis, atypical-pANCA on ethanol-fixed granulocytes appears to be a suitable serological marker of food protein-induced proctitis/proctocolitis and suggests a possible involvement of an autoimmune mechanisms in the pathogenesis of this disease.

## 1. Introduction

Over the last decade, a dramatic increase in gastrointestinal diseases has been seen in very young children. Some of them originated from an exaggerated immune response caused by various mechanisms from classical IgE mediated atopy to non-IgE mechanisms. Food protein-induced proctitis/proctocolitis is probably the most common noninfectious colitis in children in the first year of life occurring in the acute or chronic form [1–5]. The disease is frequently associated with annoyance and the child fails to thrive. Along with the overall clinical symptoms, diarrhea is the main manifestation of the disease [6–10].

Symptoms of chronic food protein-induced proctitis/proctocolitis are nonspecific, may vary depending on the extent of intestinal damage, and are associated with pathological features in the stool. The stool is characterized by occurrence of mucus, bloody mucus, or blood only. The blood—strings or dots, a larger amount appearing rarely—is only on the surface, but there should always be the presence of fresh, undigested blood. Red blood cells and polymorphonuclear leukocytes were demonstrated in loose stools, and eosinophils were also identified [11]. Food protein-induced proctitis/proctocolitis accounts for up to 80% of cases of non-IgE-mediated responses that started after delivery. Clinical manifestations may appear after the first oral contact with the causative allergen. Symptoms usually disappear after deployment of elimination diets, usually with a delay of more than 72 hours. The disease is benign and resolves by age 12–24 months in most patients. The causal food allergen can then be added back into the diet of the children. However, it is recommended to control clinical signs later (e.g., blood in the stool). The sensitization mechanism is still not completely elucidated [12–15].

Unfortunately, there is no routine noninvasive test that would be specific for food protein-induced proctitis/proctocolitis. Diagnosis is based on a family history, exclusion of other causes (infectious), laboratory tests, and mainly the positive effect of the elimination diet excluding casual foods on the patient's health condition. It is assumed that cow's milk antigens could trigger the development of food protein-induced proctitis/proctocolitis [16]. Cow's milk protein may induce the symptoms after the first feeding. The cow's milk proteins are among the first ones which the infant encounters; not all children are fully breastfed immediately after the birth. However, the disease occurs also in breastfed infants. It has been found that traces of cow's milk proteins also pass into breast milk from normal consumption of dairy products by a nursing mother.

The aim of our study was to find a noninvasive laboratory test or tests that may be helpful to contribute in the diagnosis of food protein-induced proctitis/proctocolitis.

## 2. Material and Methods

### 2.1. Patient Groups

Twenty-five children (12 boys and 13 girls, aged 1–24 months), diagnosed with food protein-induced proctitis/proctocolitis, were included in the study, and 5 boys and 3 girls had the coincidence of atopic dermatitis. 8/25 infants were fully breastfed, 8/25 children received milk hydrolyzate, 3/25 children were on egg-free diet, and 6/25 patients had no dietary restrictions (Table 1). The diagnosis was based on clinical symptoms: diarrhoea, often with mucus and/or blood, pathological signs in the stool dyspepsia, abdominal discomfort (pain and/or colic), failure to thrive, or being on the early onset of atopic eczema. Other causes of the disease were excluded (infection, rectal rhagades, and surgical complications, particularly invagination and others). Detailed family history was taken from all the parents. Inhalation allergy was found in the history of the parents in 14 cases (9 mothers and 6 fathers). Food allergy was found in two cases (mothers). One case of coeliac disease (mother) and one case of ulcerative colitis (father) were reported. Children included in the study were examined by skin prick tests focused on food allergens (cow's milk, eggs) (ALK-Abello). All the results were negative (Table 5). The positive effect of the elimination diet excluding casual foods on the patient's health condition was confirmed in all children included in patient's group. Mothers whose children were younger than 6 months were tested for ANCA IgG (IIF on the ethanol-fixed granulocytes) to eliminate potential effect of transmission of autoantibodies across the placenta. All the results were negative. The possibility of future inflammatory bowel diseases (IBD) development in these children was excluded (after 3 years of follow-up).

### 2.2. Control Group

Eighteen children (10 boys and 8 girls) aged up to 48 months were included in the control group. Their diagnoses were not related to the gastrointestinal tract. Allergic rhinitis was the main diagnosis for 12 children, and 4 children with bronchial asthma and 2 children with unspecified fever were included (Table 1).

Infant patients were treated in the Center for Food Allergy of the Pediatric Department of the Hospital Bulovka, Prague, or at the private outpatient clinic Immunoflow, Prague. All patient's sera were analyzed in the immunological laboratory of the Hospital Bulovka, Prague, or in the Department of Institute for Clinical and Experimental Medicine, Prague. All tested parameters were requested by physicians as part of routine laboratory screening. Sera of all infants were not tested for all the parameters due to lack of material. The data management met all criteria recommended by the Ethical Committee of the Hospital Bulovka.

### 2.3. Methods

#### 2.3.1. Detection of Autoantibodies

ANCA antibodies were detected by an indirect immunofluorescence (IIF) on the ethanol-fixed granulocytes. Antibodies were tested in IgA and IgG isotypes (slides and conjugates from The Binding Site, Birmingham, UK). Patient serum was diluted 1 : 10 in PBS with Tween 20 and was tested in parallel in both isotypes. Slides were read under the Nikon Eclipse E400 fluorescence microscope (Nikon Instruments Europe B.V.).

ELISA kit-ANCA-PROFILE was used to detect antibodies against the individual antigens in the IgG isotype (Euroimmun, Luebeck, Germany). The kit contains 6 different antigens, each linked to a separate well (proteinase 3, lactoferrin, myeloperoxidase, neutrophil elastase, cathepsin, and BPI-bacterial/permeability-increasing protein). Patient sera were diluted 1 : 100 in the dilution buffer according to the instructions of the manufacturer. Measurement was carried out on a Sunrise spectrophotometer (Tecan, Switzerland) at 450 nm and a reference measurement at a wavelength of 620 nm.

Antibodies against* Saccharomyces cerevisiae* (ASCA) were detected by IIF on the slides with the yeast* S. cerevisiae*. Antibodies were tested in an IgA isotype (*Saccharomyces cerevisiae* and the conjugate, Euroimmun, Luebeck, Germany). Patient sera were diluted 1 : 100 in PBS with Tween 20. Slides were read under the Nikon Eclipse E400 fluorescence microscope (Nikon Instruments Europe B.V.).

Antibodies against nuclear antigens (ANA) were detected by IIF on the slides with HEp-2 cells. Antibodies were tested in IgG isotype (HEp-2 cells and the conjugate, The Binding Site, Birmingham, UK). Patient sera were diluted 1 : 80 in PBS with Tween 20. Slides were read under the Nikon Eclipse E400 fluorescence microscope (Nikon Instruments Europe B.V.). This test was performed to eliminate the interference of ANA with ANCA, which can be interchangeable in some cases.

Antibodies against the tissue transglutaminase IgG/IgA were detected by ELISA (The Binding Site, Birmingham, UK). Measurement was carried out on a Sunrise spectrophotometer (Tecan, Switzerland) at 450 nm and a reference measurement at a wavelength of 620 nm.

#### 2.3.2. Other Immune Parameters

Concentrations of immunoglobulins were measured by nephelometry on the Image kinetic nephelometer (Beckman-Coulter, Brea, CA, USA). Serum total IgE was measured by chemoluminometry (Immulite, Siemens, USA). Serum levels of allergen-specific IgE against a mixture of inhalation allergens (Phadiatop) and food allergens (FX5: egg white, cow's milk, codfish, peanuts, soy, and wheat) were measured by the FEIA method (UniCap250, Thermo Fisher Scientifics Inc.) according to the instructions of the manufacturer. The evaluation was performed quantitatively in kU/l or qualitatively by the RAST scoring system. Calprotectin concentrations (clone MRP8/14) in serum were measured by ELISA (PhiCal Calprotectin ELISA Kit of Immundiagnostik AG, Bensheim). Measurement was carried out on a Sunrise spectrophotometer (Tecan, Switzerland) at 450 nm and a reference measurement at a wavelength of 620 nm.

## 3. Results

### 3.1. IgG and IgA Antineutrophil Cytoplasmic Antibodies (ANCA)

**Figure 1:**
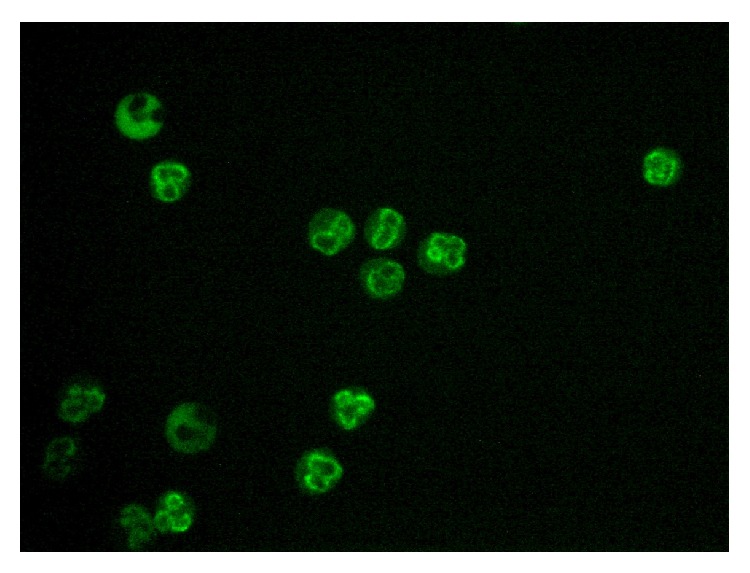
Atypical p-ANCA (serum of patient with diagnosis of food protein-induced proctitis/proctocolitis).

IgG and IgA antineutrophil cytoplasmic antibodies (ANCA) were tested by indirect immunofluorescence in the sera of 25 patients and 18 controls. ANCA IgG was detected in the sera of 24 patients. ANCA IgA was found in the sera of 5 children with food protein-induced proctitis/proctocolitis. Four patients showed the presence of antibodies in both isotypes. None of the patients included in the control group demonstrated the presence of ANCA IgG and IgA. All the positive samples showed atypical perinuclear type of fluorescence (a-pANCA). This type of fluorescence tightly surrounds the nucleus with a fluorescent cytoplasmic rim. This fluorescence type has been described frequently in adult patients with IBD and liver diseases (Table 2 and Figure 1).

With this respect antibodies against individual neutrophil cytoplasmic enzymes, ANCA profile (myeloperoxidase, proteinase 3, lactoferrin, BPI, neutrophil elastase, and cathepsin), in the IgG isotypes were tested by ELISA. In the patients group, only 4 sera were positive (2x antielastase and 2x anti-PR3). No patients showed the presence of antibodies against two or more antigens simultaneously. In the control group, no positivity test was detected (Table 3).

### 3.2. Other Autoantibodies

All patients and controls were tested for the presence of antinuclear antigens. Antinuclear antibodies (ANA) were observed by IIF on the Hep-2 cells. No ANA fluorescence was observed in the sera of 24 patients and all the controls. One patient had a weakly positive ANA (speckled fluorescence) (Table 3).

*Saccharomyces cerevisiae* IgA antibodies were also tested. No positive result was intended. Antibodies against endomysium and tissue transglutaminase, both IgG and IgA, were monitored to exclude early phases of celiac disease. No positive results were observed (Table 3).

### 3.3. Total IgG, IgA, IgM, IgE, and Specific IgE

Immunoglobulin concentrations (IgG, IgA, IgM, and IgE) were determined to complete the immune immunological profile. IgA concentrations were decreased below the normal age-adjusted range in the serum of 13 patients, increased concentrations of IgA were found in 4 patients, and 7 patients were within the normal range. Total IgE was increased in the serum of 8 patients and 13 patients had normal serum total IgE (data not shown).

Specific IgE antibodies against the FX5 mixture of food allergens were tested with respect to suspected hypersensitivity to food components. Specific IgE against food allergens were not detected in the samples of the major part of children. FX5 spec.IgE levels were increased in 2 patients, and FX5-specific IgE antibodies were detected in 2 children of the control group (Table 4).

Due to a lack of stool samples, calprotectin levels were measured in patients' sera. Only 2 out of 23 patients had elevated serum concentration. Serum calprotectin levels were not measured in the control sera due to lack of material.

## 4. Discussion

Food protein-induced proctitis/proctocolitis is characterized as an immunopathological reaction to food proteins (mostly cow's milk proteins and soy proteins, according to the geographical origin of infants). It is supposed that, in fully breastfed children, food protein-induced proctitis/proctocolitis is caused by the transfer of protein fragments from food to a breast milk [15–17]. Diagnosis is based on the family history, clinical symptoms, exclusion of the other causes of the ailment, and commitment to a diet of nursing mothers or nonbreastfed children. Signs of a food protein-induced proctitis/proctocolitis are loose stools with blood or mucus, impaired absorption, and subsequent failure of the child to thrive. Anemia is present in severe cases of food protein-induced proctitis/proctocolitis. Infectious etiology and metabolic disorders should be excluded before diagnosis.

The pathogenetic mechanism of food protein-induced proctitis/proctocolitis is yet unclear. It is supposed that non-IgE mediated immunological reaction against food antigens is involved in pathogenesis of food protein-induced colitis. The disease is often considered to be T cell mediated disorder and that T cell activation by food antigens could mediate intestinal inflammation through production of proinflammatory cytokines. Humoral responses are not well understood and in most cases independent on the production of specific IgE. However, in some cases skin tests are positive to the causal food. Leukocytosis is common finding in patients and neutrophils have been found in stool of patients. Increase in peripheral neutrophils could be explained by the overproduction of proinflammatory cytokines (e.g., TNF alfa) and chemokines. Eosinophil accumulation in intestinal tract is also commonly found in food protein-induced proctitis/proctocolitis patients and sometimes considered to be independent on allergic sensitisation. Interestingly, also the microarray analysis of mucosal biopsies specimens from neonates with rectal bleeding does not support the concept that food protein-induced colitis is caused by allergic mechanisms [18].

Recently, the influence of infectious agents such as* Streptococcus*, HIV, hepatitis, or* Aspergillus* and their peptides has been associated with the development of autoantibodies leading to ANCA-associated vasculitis as a consequence of structural resemblance between the autoantigen and peptide of infectious origin [19]. Another explanation of autoantibody presence is that the higher permeability of intestine in very young children can lead to inflammation, which might be induced by casual food (mostly cow's milk protein). This inflammation is accompanied by the disintegration of epithelial layer and influx of inflammatory cells (mostly neutrophils). This process can reveal epitopes against which autoantibodies could be synthesized. This theory is supported by a fact that around the third year of age of the child this food (cow's milk protein) can be put back into a diet. At the same time around the third year of age the autoantibodies usually disappear. Our new finding of common presence of leukocyte autoantibodies in sera of patients suggests the possible involvement of autoimmunity in pathogenic mechanism; however, the pathogenic role of these autoantibodies in the disease has not been yet studied.

The aim of our work was to find a laboratory marker that may help to distinguish food protein-induced proctitis/proctocolitis from other diseases of young children with similar clinical manifestations. 25 patients with food protein-induced proctitis/proctocolitis were included in the study. Specific IgE against basic foods (FX5) were tested in the sera of these patients. Skin tests were made with cow's milk and egg proteins. IgE mediated allergy was not confirmed in most patients. The question of whether these tests designed for older children or adults are sufficiently sensitive to determine the concentration of specific IgE antibodies in very young children is not yet answered. Our unpublished results in these patients show that “basophil activation test” with cow's milk and egg proteins might reflect the activation of basophils in some cases. This would mean that the routinely used tests are not sensitive enough to detect very low IgE serum concentrations. IgE antibodies could be bound to high affinity receptors on the surface of basophils. This question should be investigated and resolved using nonroutine procedures. Simultaneously, it should be assessed whether food-allergen-IgE may be detected later in life and whether these children develop IgE-mediated food allergy.

Since food protein-induced proctitis/proctocolitis is characterized as intestinal inflammation with infiltration by neutrophilic granulocytes, we tested ASCA and ANCA. ANCA antibodies were detected by indirect immunofluorescence on ethanol-fixed granulocytes and by ELISA methods. ASCA antibodies were negative in all the tested patients. However, a-pANCA in the isotype IgG (96%) and IgA (20%) were detected by the indirect immunofluorescence. The type of fluorescence was the same as that described for adult patients with IBD (Figure 1). The fluorescence is typically perinuclear with a nuclear rim. Generally, this type of fluorescence was described in patients with IBD, especially those with ulcerative colitis [20]. Regarding the age of children, the transfer of IgG across the placenta was suspected. However, the transmission of IgG autoantibodies from mother to a child was not confirmed since “a-pANCA” in the IgG isotype was not demonstrated in the mother's serum. Surprisingly, children aged one month were able to produce autoantibodies in response to an intense inflammation. Five children from this group were observed after the first finding of “a-pANCA.” We found that with increasing age and the gradual decline of clinical symptoms, the intensity of fluorescence on the ethanol-fixed granulocytes decreased (data not shown). Antibodies against neutrophil elastase (2 patients) and PR3 (2 patients) were only detected. These results are in agreement with previously published data [21, 22]. Terjung et al. suggest that a-pANCA are directed against 50-kilodalton myeloid-specific nuclear envelope protein [23]. On the other hand, there is an evidence that they might be antibodies against lactoferrin, which cannot be identified by routinely used methods [24]. Recently, it was found that anti-PR3 antibodies, which were measured by the new chemiluminescent immunoassay (CLIA), were detected in patients with IBD, especially with UC [25]. Another study suggests that an ethnic basis (Indian origin) may influence the tendency to the response by synthesis of anti-PR3 in UC patients. These IBD pediatric patients with the rare association of PR3 antibodies did not show any evidence of systemic or local vasculitis [26]. It is obvious that the target antigen has not yet been fully identified and the development of new sensitive methods can yield new insights into this issue. Although the evaluation of positive samples in indirect immunofluorescence is always affected by subjective experience and quality of the preparation, the detection of  “a-pANCA” in indirect immunofluorescence on ethanol-fixed granulocytes is still a standard method.

To avoid misinterpretation of simultaneous ANCA and ANA positivity, all patients were tested on Hep-2 cells and only one patient showed the presence of ANA antibodies.

Our results of ANCA positivity and ANA, ASCA, a-EMA, and a-tTg negativity and negativity of specific IgE against food allergens correspond with previously published results of Nevoral et al., who focused on the link between food protein-induced proctitis/proctocolitis and changes in the microbial intestinal flora. This group found that the number of anaerobic bacteria (*Bifidobacterium* and* Lactobacillus*) was significantly higher in healthy individuals.* Clostridium* was in contrast detected only in the stool of* Bifidobacterium* negative infants [27]. Protection against potentially harmful substances is ensured by a number of factors, including microbiota, which plays an important role in mucosal physiology, participates in mucosal barrier function, and thus regulates immunological and immunopathological reactions. The gradual maturation of the mucosa leads to improvement of its protective functions and gradual extinction of inflammation. The development of mucosal immune system might be modulated also by the presence of polyreactive natural IgA autoantibodies found in human colostrums [28, 29].

The fluorescence of “a-pANCA” on ethanol-fixed granulocytes of children with food protein-induced proctitis/proctocolitis closely resembled the fluorescence pattern detected in IBD patients. Several articles describe the coexistence of food protein-induced proctitis/proctocolitis with IBD or subsequent development of IBD in “food protein-induced proctitis/proctocolitis” patients. Patients enrolled in this study were followed up until three years of age. So far, none of these children have developed either ulcerative colitis or Crohn's disease. Children included in our patient's group were under the age of 2 years with the predominance of children under 6 months at the first examination. This may be the reason for the absence of other diagnoses and other autoantibodies (like ASCA). It is also possible that the length of the monitoring of these children was not long enough and that this disease may develop in the adolescence as described [30].

We measured also the concentration of serum calprotectin. Two patients had elevated levels of calprotectin. The correlation between serum calprotectin and disease activity in patients with IBD was described only by some authors [31], and others have not found such a relationship [32]. Unfortunately, we were not able to test stool, where the amount of calprotectin significantly increases before the relapse of IBD [33]. The question of whether fecal calprotectin would be a suitable noninvasive marker of food protein-induced proctitis/proctocolitis thus remains open.

In conclusion, from food protein-induced proctitis/proctocolitis parameters tested, “a-pANCA” on the ethanol-fixed granulocytes appeared to be a suitable marker for confirming the diagnosis. This fluorescence pattern is typical for IBD (ulcerative colitis) and autoimmune hepatopathy in adults. We have demonstrated “a-pANCA” positivity in a completely different group of infants whose disease is similarly accompanied by intense intestinal inflammation with participation of neutrophil granulocytes. The association of autoantibody positivity with food protein-induced proctitis/proctocolitis indicates the possible involvement of autoimmune mechanisms in pathogenesis of this disease.

## Figures and Tables

**(a):** 

Diagnosis, patient group		Diagnosis, control group	
Food protein-induced proctitis/proctocolitis	25	Asthma	4
Atopic eczema	8	Allergic rhinitis	12
Others^*∗*^	1	Others^*∗∗*^	2

^*∗*^Others: vomiting, ^*∗∗*^others: fever.

**(b):** 

Diet, patient group		Diet, control group	
Breastfed	8	Without dietary restriction	18
Milk hydrolyzate	5		
Amino acids	3		
Egg-free diet	3		
Without dietary restriction	6		

**Table:** (a) Patient group

	IIF IgG	IIF IgA
Negative	1	20
Positive	24	5

**Table:** (b) Control group

	IIF IgG	IIF IgA
Negative	18	18
Positive	0	0

**(a):** 

Patient group	Method	Negative	Weekly positive	Positive	Strongly positive
a-pANCA IgG	IIF	1	3	14	7
a-pANCA IgA	IIF	20	0	3	2
ANCA profile IgG	ELISA	21	2 (2x PR3)	2 (2x ELA)	0
ANA IgG	IIF	24	0	1 (granular)	0
ASCA IgA	IIF	25	0	0	0
a-ENDO IgA	IIF	10	0	0	0
a-ENDO IgG	IIF	10	0	0	0
a-tTg IgA	ELISA	13	0	0	0
a-tTg IgG	ELISA	13	0	0	0

**(b):** 

Control group	Method	Negative	Weekly positive	Positive	Strongly positive
a-pANCA IgG	IIF	18	0	0	0
a-pANCA IgA	IIF	18	0	0	0
ANCA profile IgG	ELISA	18	0	0	0
ANA IgG	IIF	18	0	0	0
ASCA IgA	IIF	18	0	0	0
a-ENDO IgA	IIF	18	0	0	0
a-ENDO IgG	IIF	18	0	0	0
a-tTg IgA	ELISA	18	0	0	0
a-tTg IgG	ELISA	18	0	0	0

**(a):** 

Patient group	Method	Normal	Reduced	Increased
Total IgA	Nephelometry	7	13	4
Total IgG	Nephelometry	16	5	2
Total IgM	Nephelometry	14	6	3
Total IgE	Nephelometry	13	0	8
spec.IgE Phadiatop	FEIA	16	—	0
spec.IgE FX5	FEIA	18	—	2
Calprotectin	ELISA	21	0	2

**(b):** 

Control group	Method	Normal	Reduced	Increased
Total IgA	Nephelometry	6	10	2
Total IgG	Nephelometry	12	6	0
Total IgM	Nephelometry	10	6	2
Total IgE	Nephelometry	8	—	10
spec.IgE Phadiatop	FEIA	8	—	6
spec.IgE FX5	FEIA	12	—	4

**Table 5:** Major features of the 25 infants with Food protein-induced proctitis/proctocolitis.

Patient (sex)	Age month	Diet	dg. parents	a-pANCA IgG IIF	a-pANCA IgA IIF	ANA IgG IIF	ANCA profile ELISA	ASCA IIF	a-EMA IgA	a-EMA IgG	a-tTg IgA	a-tTg IgG	Prick tests cow's milk egg	Phadiatop MJ/mL	FX5 MJ/mL	Calprotectin ng/mL <3000 ng/mL
1 (M)	6	Milk hydrolyzate	—	**Positive**	Neg.	Neg.	Neg.	Neg.	—	—	1	1	Negative	—	<0.35	—
2 (F)	2	Breastfeeding	Mother IA	*Highly positive*	**Positive**	Neg.	Neg.	Neg.	Neg.	Neg.	0.5	1	Negative	—	<0.35	390
3 (F)	5	Milk hydrolyzate	Mother IA	*Highly positive*	Neg.	Neg.	Neg.	Neg.	—	—	—	—	Negative	<0.35	<0.35	339
4 (M)	2	Breastfeeding	Mother IA	*Highly positive*	Neg.	Neg.	**Positive** **ELAST**.	Neg.	—	—	—	—	Negative	<0.35	<0.35	511
5 (F)	7	Milk hydrolyzate	—	*Highly positive*	Neg.	**Positive (granular)**	Neg.	Neg.	—	—	—	—	Negative	—	—	*4286*
6 (F)	24	Egg-free diet	Mother IA	**Positive**	Neg.	Neg.	Neg.	Neg.	Neg.	Neg.	1.2	1	Negative	2.22	<0.35	*4451*
7 (F)	1	Breastfeeding	—	**Positive**	Neg.	Neg.	***Weakly positive PR3***	Neg.	Neg.	Neg.	0.5	0.9	Negative	<0.35	<0.35	677
8 (M)	1	Breastfeeding	Father IA	***Weakly positive***	Neg.	Neg.	Neg.	Neg.	—	—	—	—	Negative		<0.35	385
9 (M)	24	—	Mother IA	**Positive**	Neg.	Neg.	Neg.	Neg.	—	—	—	—	Negative	<0.35	<0.35	—
10 (M)	2	Breastfeeding	—	**Positive**	Neg.	Neg.	Neg.	Neg.	—	—	—	—	Negative	—	—	—
11 (M)	3	Breastfeeding	Mother, father IA	**Positive**	Neg.	Neg.	Neg.	Neg.	—	—	—	—	Negative	—	<0.35	83
12 (F)	24		Mother IA, FA	***Weakly positive***	Neg.	Neg.	Neg.	Neg.	Neg.	Neg.	1.1	1	Negative	<0.35	<0.35	620
13 (F)	7	Milk hydrolyzate	Mother IA, FA	**Positive**	Neg.	Neg.	Neg.	Neg.	—	—	—	—	Negative	<0.35	<0.35	376
14 (M)	23	—	—	**Positive**	*Highly positive*	Neg.	***Weakly positive*** ***PR3***	Neg.	Neg.	Neg.	2.69	1.1	Negative	—	<0.35	641
15 (F)	24	—	Mother IA	**Positive**	**Positive**	Neg.	**Positive** **ELAST.**	Neg.	Neg.	Neg.	1	1.2	Negative	<0.35	<0.35	1014
16 (M)	4	Milk hydrolyzate	—	**Positive**	Neg.	Neg.	Neg.	Neg.	—	—	1.1	1.1	Negative	<0.35	<0.35	510
17 (F)	18	Egg-free diet	Father IA	**Positive**	Neg.	Neg.	Neg.	Neg.	—	—	1.1	1.2	Negative	—	—	548
18 (M)	15	—	Father IA	Neg.	**Positive**	Neg.	Neg.	Neg.	Neg.	Neg.	1.1	1	Negative	<0.35	<0.35	1270
19 (F)	18	—	Mother celiakie	*Highly positive*	Neg.	Neg.	Neg.	Neg.	Neg.	Neg.	1	1	Negative	<0.35	<0.35	1784
20 (F)	6	Milk hydrolyzate	—	*Highly positive*	*Highly positive*	Neg.	Neg.	Neg.	Neg.	Neg.	1	1.1	Negative	—	—	821
21 (F)	4	Milk hydrolyzate	Father IA	**Positive**	Neg.	Neg.	Neg.	Neg.	Neg.	Neg.	0.5	1.9	Negative	<0.35	*0.97*	340
22 (M)	6	Milk hydrolyzate	Father ulcerative colitis	**Positive**	Neg.	Neg.	Neg.	Neg.	—	—	—	—	Negative	—	*>100*	443
23 (M)	15	Egg-free diet	—	**Positive**	Neg.	Neg.	Neg.	Neg.	—	—	—	—	Negative	—	—	952
24 (F)	1	Breastfeeding	—	*Highly positive*	Neg.	Neg.	Neg.	Neg.	—	—	—	—	Negative	<0.35	—	454
25 (M)	1	Breastfeeding	Father IA	***Weakly positive***	Neg.	Neg.	Neg.	Neg.	—	—	—	—	Negative	<0.35	<0.35	980

IA: inhalation allergy.

FA: food allergy.

## Abbreviations

a-EMA: Antiendomysium antibodies
a-MPO: Antimyeloperoxidase antibodies
ANA: Antinuclear antibodies
a-pANCA: Atypical p antineutrophil cytoplasmic antibodies
a-PR3: Antiproteinase 3 antibodies
ASCA: Anti-*Saccharomyces* cerevisiae antibodies
a-tTg: Antitissue transglutaminase antibodies
BPI: The bactericidal/permeability-increasing protein
Elast.: Elastase
ELISA: Enzyme-linked immunosorbent assay
FEIA: Fluorescence enzyme immunoassay
IBD: Inflammatory bowel disease
IIF: Indirect immunofluorescence
MPO: Myeloperoxidase
PR3: Proteinase 3.
